# Molecular Role of EGFR-MAPK Pathway in Patchouli Alcohol-Induced Apoptosis and Cell Cycle Arrest on A549 Cells* In Vitro* and* In Vivo*

**DOI:** 10.1155/2016/4567580

**Published:** 2016-10-17

**Authors:** XinGang Lu, Liu Yang, ChengHua Lu, ZhenYu Xu, HongFu Qiu, JiaJia Wu, JingWen Wang, JiaFeng Tong, Yin Zhu, Jie Shen

**Affiliations:** ^1^Department of Traditional Chinese Medicine, Huadong Hospital, Fudan University, Shanghai 200040, China; ^2^Department of Oncology, Baoshan Hospital of Integrated Traditional Chinese and Western Medicine, Shanghai University of Traditional Chinese Medicine, Shanghai 201999, China; ^3^Department of Respiratory, Longhua Hospital, Shanghai University of Traditional Chinese Medicine, Shanghai 200030, China; ^4^Department of Traditional Chinese Medicine, Wujing Hospital, Shanghai University of Traditional Chinese Medicine, Shanghai 200241, China; ^5^Department of Rehabilitation, The Second People Huaian Hospital, Xuzhou Medical College, Huaian 223002, China; ^6^Department of Oncology, Huadong Hospital, Fudan University, Shanghai 200040, China; ^7^Department of Pharmacy, Huadong Hospital, Fudan University, Shanghai 200040, China

## Abstract

Nowadays, chemotherapy is still the main effective treatment for cancer. Herb prescriptions containing* Pogostemon cablin Benth* (also known as “Guang-Huo-Xiang”) have been widely used in Chinese medicine today. In our research, we found that patchouli alcohol, a compound isolated from the oil of* Pogostemon cablin Benth*, exerted antitumor ability against human lung cancer A549 cells ability both* in vitro* and* in vivo*. MTT assay was used to assess cell viability. Hoechst 33342 staining and TUNEL cover glass staining provided the visual evidence of apoptosis. Caspase activity measurement showed that patchouli alcohol activated caspase 9 and caspase 3 of mitochondria-mediated apoptosis. Consistently, patchouli alcohol inhibited the xenograft tumor* in vivo*. Further investigation of the underlying molecular mechanism showed that MAPK and EGFR pathway might contribute to the antitumor effect of patchouli alcohol. Our study proved that patchouli alcohol might be able to serve as a novel antitumor compound in the clinical treatment of lung cancer.

## 1. Introduction

*Pogostemon cablin Benth*, also known as Guang-Huo-Xiang in China, is a medicinal plant widely planted in Philippines, Malaysia, India, and China [1]. In the clinical practice of traditionally Chinese medicine, Guang-Huo-Xiang has been widely used in numerous prescriptions including Lianhua-Qingwen capsule [2]. Additionally, the patchouli oil, a nature perfume from Guang-Huo-Xiang, has been widely used to resolve damp, improve appetite, arrest vomiting, and dispel summer-heat as well as summer-damp [3–5] for a long time in history since ancient times in China.

**Figure 1:**
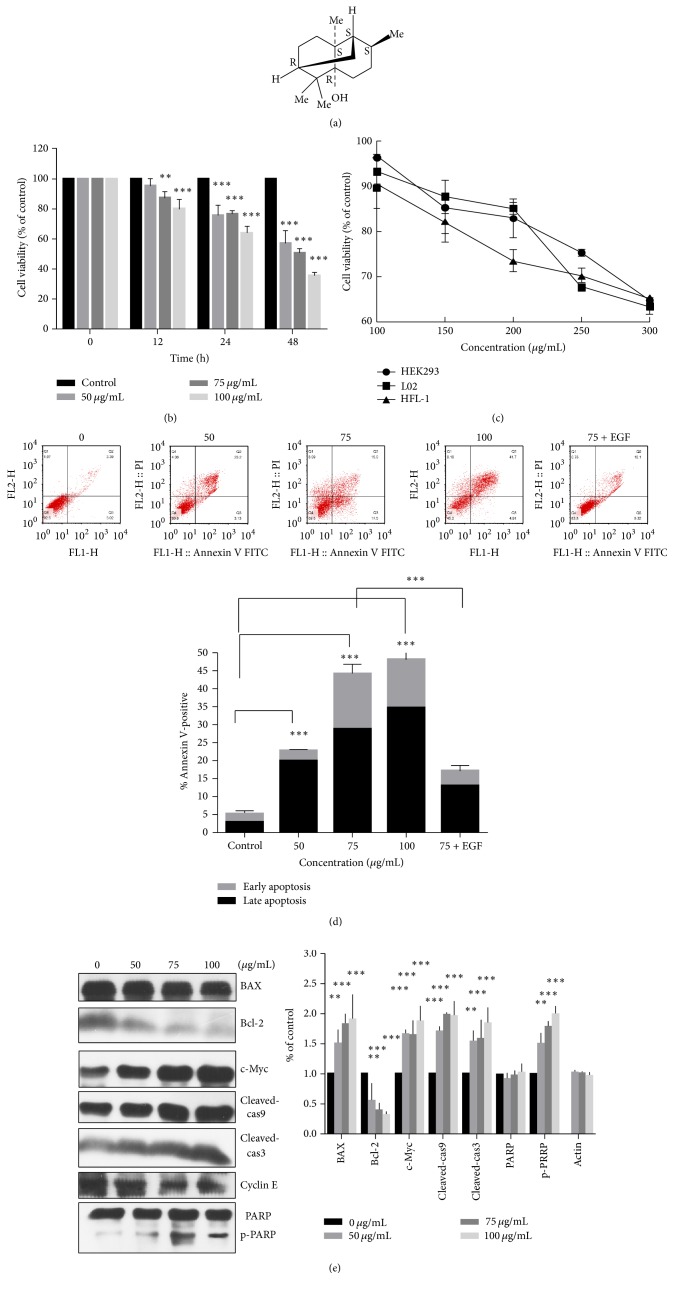
Growth inhibition and apoptosis effect of PA. (a) The chemical structure of PA. (b) Inhibition effect of PA on A549 cells using MTT assay. (c) Effect of PA on HEK293, L02, and HFL-1 cells for 48 h using MTT assay. (d) The increasing tendency of apoptotic ratio induced by increasing PA concentrations of 0, 50, 75, and 100 *μ*g/mL and 75 *μ*g/mL combined with EGF. (e) Mechanism of PA induced apoptosis on A549 cells. A549 cells were exposed for 48 h with increasing PA. Proteins levels were probed by western blotting assay. Statistical differences were compared between PA treatment group and control group and considered significant at the levels of ^*∗∗*^ *P* < 0.01 or ^*∗∗∗*^ *P* < 0.001.

It has been found that patchouli alcohol (PA) is the main effective component of Guang-Huo-Xiang, which accounts for 20–26% weight of the patchouli oil [6]. The chemical structure of PA is shown in Figure 1(a). PA is used for the quality control objective of patchouli oil in pharmaceutical industry (The Pharmacopoeia Commission of PRC 2010). Accumulating reports have demonstrated that PA had multiple effects such as anti-inflammatory [7, 8], antivirus [9–12], antimicrobial [13–17], and insecticidal [18, 19], protective effect on fluidity of intestinal epithelial cells [20], protective effect on acute lung injury [21], radical-scavenging activity [22], inhibitory activity on platelet-activating factor (PAF) activation [23], and antiemetic activity [24].

Recently, several studies have revealed the anticancer activity of PA and the possible underlying mechanisms. PA suppressed cell proliferation and induced apoptosis in a concentration-dependent manner in two human colorectal cancer cell lines (HCT116, SW480). Moreover, PA suppressed cell growth in MCF7, BxPC3, PC3, and HUVEC cells. PA treatment to HCT116 and SW480 cells triggered p21 expression and inhibited the expressions of Cyclin D1 and Cyclin-dependent kinase 4 (CDK4). Meanwhile, PA attenuated the expressions of histone deacetylases (HDACs) and c-myc, in two human colorectal cancer cells. PA treatment also resulted in the transcriptional activity of NF-*κ*B via an increase of p65 nuclear translocation [25]. Additionally, PA inhibited the proliferation of HeLa cells* in vitro* [26].

However, the anticancer ability on human lung cancer of PA has not been investigated. In this study, we found that PA was able to suppress the growth of A549 cells both* in vitro* and* in vivo*. Treatment of PA induced apoptosis in and arrested the cell cycle progression of A549 cells. Further investigation on the molecular mechanism showed that PA inhibited the phosphorylation of EGFR and thereby blocked the downstream signaling, which may be responsible for the anticancer activity of PA. Our study proved that PA might serve as a potential compound for the treatment of non-small-cell lung cancer in the clinical practice and provided more insights for developing novel anticancer agents from medicinal plants.

## 2. Materials and Methods

### 2.1. Materials

Pancreatin, penicillin, RPMI-1640 medium, Fetal Bovine Serum (FBS), Phosphate Buffered Saline (PBS), and streptomycin were supplied from Gibco (Gibco, Carlsbad, USA); A549, L02, HEK293, and HFL-1 cells were gained from the Cell Bank of Chinese Academy of Sciences (Shanghai, China). Terminal Deoxynucleotidyl Transferase- (TdT-) mediated dUTP nick end labeling (TUNEL) Apo-Green Detection Kit was supplied by Roche (Roche, Basel, Switzerland), while Annexin V&PI Kit was purchased by Biotool (Selleck Chemicals, Houston, Texas, USA). Protein extraction kit, RNA extraction kit, BCA Protein Assay Kit, caspases 3 and 9 activity kit, Propidium Iodide (PI), dimethylsulfoxide (DMSO), 4′,6-diamidino-2-phenylindole (DAPI), and Hoechst 33243 were purchased from Beyotime Institute of Biotechnology (Beyotime, Haimen, China). PA standard preparation (purity > 98%) was purchased from National Institute for the Control of Pharmaceutical and Biological Products (Beijing, China). JC-10 Mitochondrial Membrane Potential Assay Kit was obtained by AAT Bioquest (AAT Bioquest, CA, USA). ECL Advanced Detection Kit was provided by Thermo Fisher (Thermo Fisher, Waltham, USA). 3-(4,5-Dimethylthiazol-2-yl)-2,5-diphenyl-2H-tetrazolium bromide (MTT) and albumin from bovine serum (ABS) were purchased from Sigma-Aldrich (St. Louis, USA). Desired primary antibodies, HRP-labeled secondary anti-mouse/anti-rabbit antibodies, were provided by Cell Signaling Technology (CST, Beverly, USA). All other unmentioned chemical reagents were of analytic grade.

### 2.2. Cell Culture and PA Treatment

A549 carcinoma cell lines and three types of normal cell lines (HEK293, L02, and HFL-1) were cultured in a humidified atmosphere of 5% CO_2_ with RPMI-1640 medium containing 10% fetal calf serum and antibiotics (100 IU/mL penicillin and 100 IU/mL streptomycin); PA standard was dissolved in DMSO for further experiment. A549 cells were treated with increasing concentrations of PA for desired hours in PA exposure groups. The control group was conducted with the same volumes of DMSO under the same conditions.

### 2.3. Cytotoxicity and Cell Viability Analysis

Cytotoxicity of PA on carcinoma and normal cells was analyzed using the MTT assay. The A549, HEK293, L02, and HFL-1 cells were seeded in culture plates and exposed to PA by increasing concentrations. Concisely, 5 × 10^3^ cells were cultured with 200 *µ*L culture medium and 20 *µ*L MTT (5 mg/mL) for 4 hours. The absorbance at 570 nm was assessed using a microplate reader (Perkin-Elmer Inc., Waltham, USA) after removing the supernatant and dissolving the formazan in DMSO. Cell viability of PA-treated cells and control cells was estimated as (absorbance of PA-treated group/absorbance of control) × 100%.

### 2.4. Hoechst 33342 Staining

The NSCLC A549 cells were cultured and treated by PA as described above. The NSCLC A549 cells were stained with Hoechst 33342 (5 *µ*g/mL) at room temperature for 5 minutes in the dark. Stained cells were observed by an inverted immunofluorescence microscopy (D5100, Nikon, Tokyo, Japan).

### 2.5. TUNEL Staining

DNA fragmentation in apoptotic cells induced by anticarcinoma drugs can be detected by TUNEL staining assay according to the manufacturer's instruction. The NSCLC A549 cells after PA treatment and control were fixed by TUNEL and DAPI. We analyzed the PA-treated group and control cells immediately using immunofluorescence microscopy described above to view the green (TUNEL) at 520 nm and the blue (DAPI) at 460 nm.

### 2.6. Flow Cytometry for Cell Cycle and Apoptosis Measurement

To detect the cell cycle and apoptotic subpopulation in PA-treated A549 cells, the A549 cells were cultured and induced by PA as mentioned. PA-treated A549 cells were harvested, stained with Annexin-FITC/PI, and measured using a FACS Calibur cytometer (BD, Biosciences, San Jose, CA, USA) according to the manufacturer's instruction. As for the investigation of cell cycle progression, PA-treated cells were harvested, fixed, and stained with PI according to the manufacture's instruction and then measured by the FACS Calibur cytometer.

### 2.7. Mitochondrial Membrane Potential (MMP)

AAT JC-10 assay kit was used to measure the loss in mitochondrial membrane potential of PA-treated A549 cells. PA-treated A549 cells were stained according to the manual and then the fluorescence was measured using a microplate reader (Perkin-Elmer Inc., Waltham, USA). The MMP was indicated by the ratio of the green fluorescence absorbance at 525 nm to the red fluorescence absorbance at 590 nm.

### 2.8. Caspases 3 and 9 Activity Assay

Caspases 3 and 9 activities were determined by using caspases 3 and 9 activity kit according to the kit instruction manual. Release of p-nitroanilide (pNA) was determined by absorbance at 405 nm using a microplate reader (Perkin-Elmer Inc., Waltham, USA). The caspase activity was demonstrated as the ratio of PA-treated cells/control A549 cells.

### 2.9. *In Vivo* Study

We conducted all the animal experiments according to the Declaration of Helsinki and the Guide for the Care and the Use of Laboratory Animals as adopted and promulgated by the United States National Institutes of Health. The animal study was licensed by the Institutional Animal Care and Use Committee of Fudan University. The 6-week-old BALB/C nude mice (Shanghai Slac Laboratory Animal) were injected with A549 cells intraperitoneally with 1 × 10^7^ cells per mouse. There were 4 randomly divided groups (6 mice each group): control group (vehicle) and PA treatment group with low, medium, and high PA dose. PA standard crystal was dissolved in saline containing 5% DMSO. When the volume of the tumor reached 100 mm^3^, 200 *µ*L 5% DMSO/saline solutions including increasing dose of PA were injected intraperitoneally into the right flank of nude mice in PA treatment group. The disinfecting saline in same volumes containing 5% DMSO was administrated into the vehicle group. PA was injected into all the PA treatment groups every 3 days. After the last treatment, the nude mice were put to death in 24 h. Tumor xenografts removed from mice were measured and fixed for next experiment. Tumor sizes were evaluated every 3 days using micrometer calipers, and tumor weights were determined at last.

### 2.10. Immunohistochemistry

The A549 xenografted tumor samples were stained using cleaved-caspase 3 (1 : 100) and Ki67 (1 : 150) antibodies separately for immunohistochemistry. Images were recorded with a fluorescence microscope (D5100, Nikon, Tokyo, Japan).

### 2.11. Western Blotting

The levels of apoptotic proteins were analyzed by western blotting analysis. Proteins of PA-treated cells and control were extracted by the extraction kit; protein concentrations were measured using BCA protein assay kit. The proteins were subjected to SDS-PAGE and electrophoretically transferred to PDVF membrane (Millipore Corp, Bedford, MA, USA). The membranes were incubated with desired primary antibodies (1 : 1000) and incubated with secondary antibody (1 : 5000) conjugated with peroxidase subsequently. These signals were then detected by a detection system (BD Biosciences, San Jose, CA, USA). The *β*-actin antibody was employed as control.

### 2.12. Statistical Analysis

Triplicate experiments were conducted with independent samples. The data were represented as mean ± SD. Statistical intergroup differences were evaluated using one-way ANOVA followed by* post hoc* Bonferroni test and were represented as ^*∗*^ *P* < 0.05, ^*∗∗*^ *P* < 0.01, or ^*∗∗∗*^ *P* < 0.001 level. All statistical analyses were performed using SPSS 19.0 (Chicago, IL, USA).

## 3. Results

### 3.1. Effect of PA Antigrowth Capability in Four Cells

We first investigated whether PA treatment could inhibit the growth of non-small-cell lung cancer cells. The A549 is most common cell model used for multiple anti-lung cancer research [27]. In our results, it was shown that PA suppressed the growth of A549 cells in a concentration- and time-dependent manner (Figure 1(b)). Lower viability was observed in A549 cells treated with PA for 48 h. The IC_50_ value of PA on A549 cells was 79.80 ± 4.09 *μ*g/mL. Further, we determined the cytotoxicity of PA in human normal cells: L02, HEK293, and HFL-1. However, more than 60% of cells were still viable when treated with PA of the concentration up to 300 *μ*g/mL (Figure 1(c)). Taken together, these data suggested that PA might serve as a potent and safe anticancer agent against non-small-cell lung cancer.

### 3.2. Effects of PA on Cell Apoptosis* In Vitro*

The best performances based on MTT results indicate that 48 h was best experiment time. PA exposure at 50–100 *μ*g/mL at 48 h treated cells showed a marked increase by Annexin V/PI assay shown in Figure 1(d). The apoptotic population in PA-treated A549 cells increased in a dose-dependent manner.

**Figure 2:**
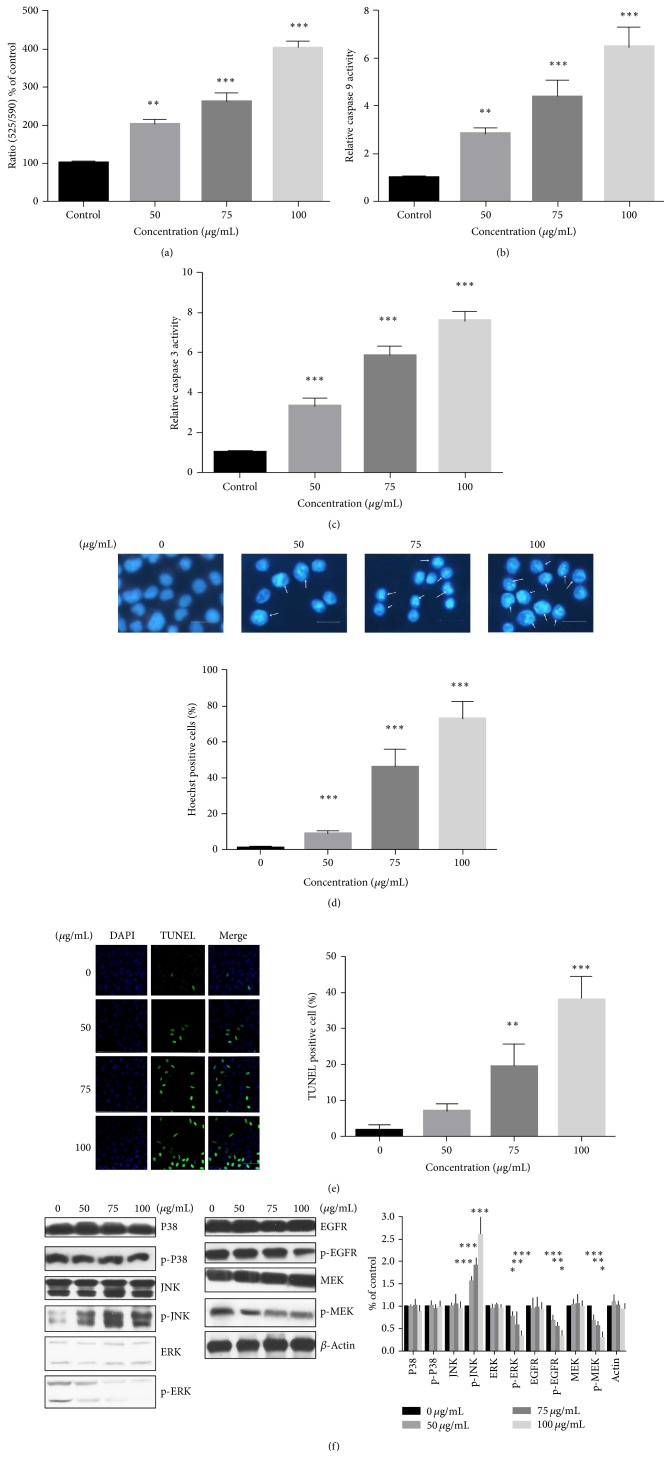
The apoptosis effect and involved signaling pathway activity of PA on A549 cells. The A549 cells were exposed for 48 h with increasing PA. (a) PA induced MMP collapse for 48 h treatment. (b) PA exposure induced caspase 9 activation in A549 cells for 48 h. (c) PA exposure induced caspase 3 activation in A549 cells for 48 h. (d) The Hoechst staining immunofluorescence microscopy image of A549 cells for Hoechst* in vitro*; Scale Bar: 10 *μ*m. (e) The TUNEL staining immunofluorescence microscopy image of A549 cells for DAPI (blue), TUNEL FITC (green), and their merge* in vitro*; Scale Bar: 50 *μ*m. (f) Effect of PA on EGFR and MAPK signaling pathways in A549 cells. Statistical differences were compared between PA treatment group and control group and considered significant at the levels of ^*∗*^ *P* < 0.05, ^*∗∗*^ *P* < 0.01, or ^*∗∗∗*^ *P* < 0.001.

We further hypothesized that PA might induce apoptosis via the mitochondrial pathway. Firstly, western blotting assays showed prompted cleavage of PARP and caspases 3 and 9 in PA-treated cells. Additionally, PA inhibited the expression of Bcl-2 but had little effect on the level of Bax, which led to decreased ratio of Bcl-2/Bax (Figure 1(e)). Consistent with above result, JC-10 assays determine the mitochondrial outer membrane permeabilization induced by PA treatment. Most anticancer agents induce collapse of MMP mainly in the mitochondria apoptotic early phase. In our present study, 24 h was conducted as PA treatment time to view the loss of MMP. It was found that PA treatment increased the ratio of green/red fluorescence of stained cells, which indicated a loss of MMP (Figure 2(a)). Activity assays revealed caspases 3 and 9 activation in the PA induced A549 cells (Figures 2(b) and 2(c)). These results suggested that induction of mitochondrial apoptosis was, at least partly, responsible for the cytotoxicity of PA in A549 cells.

Additionally, the apoptosis elicited by PA exposure was observed visually as well. Hoechst staining exhibited image results with shrinkage, nuclear fragmentation, and chromatin compaction of PA-treated cells, indicating apoptosis induction as well (Figure 2(d)). Consistently, TUNEL staining demonstrated that PA at the concentration of 50–100 *μ*g/mL led to significantly increased population of apoptotic cells (green, Figure 2(e)).

Western blotting of the PA treatment A549 versus control showed the apoptosis-related signal pathway. In present survey, we found that the phosphorylation of EGFR, a critical tyrosine kinase in the genesis and development of numerous human cancers, was suppressed by PA treatment. The EGFR phosphorylation level, containing RAS/RAF/MEK/ERK, is blocked with the increasing concentration of PA as well. The MAPK signaling pathway activities (JNK, ERK, and P38) were also evaluated after PA treatment. As shown in Figure 2(f), western blotting indicated that phosphorylation of ERK (which lies downstream of EGFR) was downregulated. However, PA caused the increasing of p-JNK while showing no impact on p-P38. The phosphorylation activation of JNK signaling pathway and downphosphorylation of EGFR pathway induced the MMP loss. These caused the unbalance of Bcl-2/Bax which elicits the following mitochondrial apoptosis pathways: releasing of c-Myc and activation of caspase 9 and caspase 3 subsequently.

### 3.3. Effects of PA on the Cell Cycle Arrest* In Vitro*

**Figure 3:**
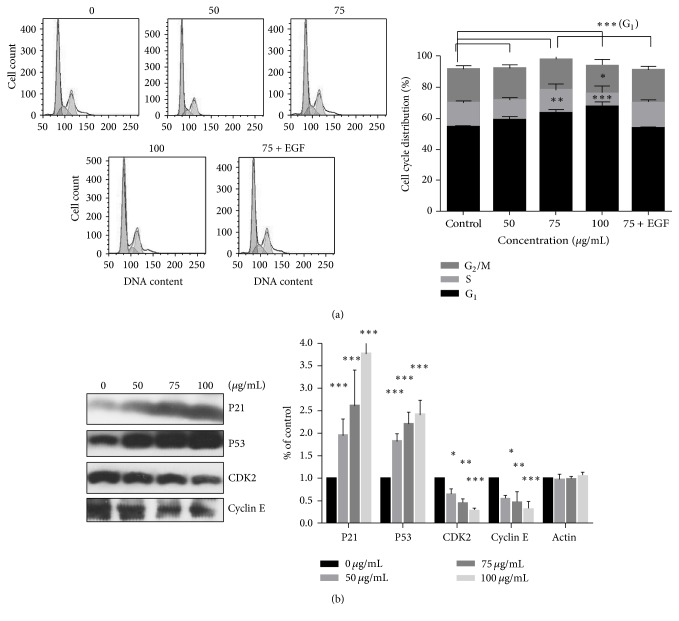
Typical G_1_/S cell cycle arrest on A549 cells induced by increasing PA exposure of 0, 50, 75, and 100 *μ*g/mL. (a) PA induced G_1_/S cell cycle arrest. (b) The involved proteins activities. Statistical differences were compared between PA treatment group and control group and considered significant at the levels of ^*∗*^ *P* < 0.05, ^*∗∗*^ *P* < 0.01, or ^*∗∗∗*^ *P* < 0.001.

We conducted cell cycle assay to evaluate the PA's impact on A549 using PI. Meanwhile, we performed western blotting to reveal the underlying mechanism.  Figure 3(a) manifested an accumulating cell cycle progression in G_1_/S phase in a dose-dependent manner in PA-treated A549 cells. Then, we determined the PA's impact on the expression of P53, P21, Cyclin E, and Cyclin-dependent kinase 2 (CDK2). Western blotting demonstrated that the expression level of P53 was activated in a dose-dependent manner and an appreciable downregulation of Cyclin E and CDK2 subsequently (Figure 3(b)).

### 3.4. Antiproliferation Effect of PA on A549 Nude Xenograft Tumor

**Figure 4:**
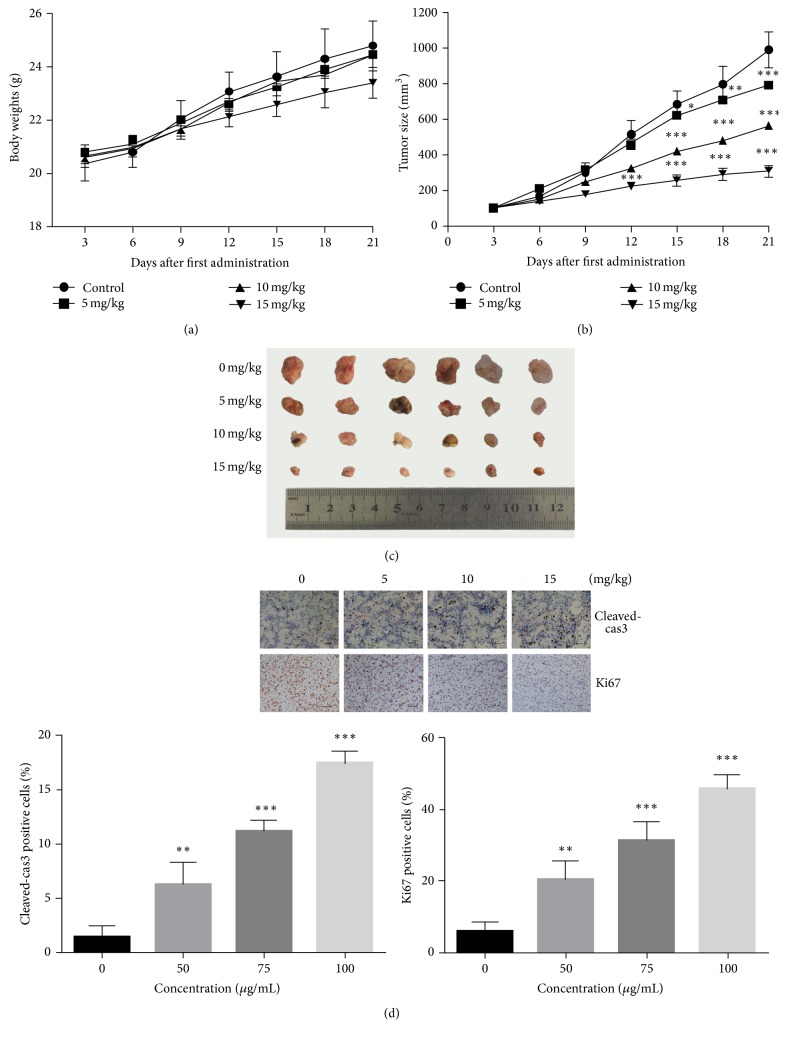
The antiproliferation effect of PA on A549 xenograft model. (a) The body weights of nude A549 models* in vivo*. (b) The tumor sizes of A549 nude models* in vivo*. (c) The tumor of each group. (d) Effect of PA on the expression levels of cleaved-caspase 3 and Ki67 in A549 nude model; Scale Bar: 100 *μ*m. Statistical differences were compared between PA treatment group and control group and considered significant at the levels of ^*∗*^ *P* < 0.05, ^*∗∗*^ *P* < 0.01, or ^*∗∗∗*^ *P* < 0.001.

We performed a study* in vivo* to investigate the antiproliferation effect of PA on A549 nude xenograft tumor. There were few changes in mice body weight in the experiment, as shown in Figure 4(a). This implied the relative safety of the injection of PA to the nude mice. After 21 days, tumors treated with PA grew smaller than control, as shown in Table 1 and Figures 4(b) and 4(c). The significantly decreasing levels of Ki67-positive cells and the increasing levels of cleaved-caspase 3 induced by the PA treatment were found in the study* in vivo* as shown in Figure 4(d). The study* in vivo* demonstrated the effects of apoptosis and antiproliferation effect of PA as well.

## 4. Discussion

*Pogostemon cablin Benth* possessed multiple pharmacology activities in previous reports [5]. PA is the main biological active constituent of* Pogostemon cablin Benth*. Published studies had shown that PA inhibited the proliferation of several carcinoma cells and normal cells such as HUVEC [25]. However, higher IC_50_ in our study demonstrated tissue specific effect of PA on HEK293 (normal kidney cell), L02 (normal liver cell), and HFL-1. Exposure to PA caused apoptosis and cell cycle arrest in A549 in a time- and dose-dependent manner. Therefore, PA may be a candidate antitumor agent although the administration or the structure of PA may call for new efforts in future.

Scientists paid attention to the drug's antiproliferation effect and intrinsic mechanism during the past decades [28–30]. Antiproliferation mechanisms are commonly associated with apoptosis and cell cycle arrest [31]. Firstly, we observed that the antiproliferation is activated by apoptosis through TUNEL and Hoechst staining. Using flow cytometry analysis, the apoptosis induced by PA was validated by Annexin V/PI assay. Apoptosis by caspase activations has two main types: caspase 8 activation is linked to extrinsic apoptotic pathway downstream death receptor like TNF-*α* receptor while caspase 9 activation is involved in the intrinsic mitochondrial apoptotic pathways [32]. Anticancer drugs induce caspase 9 activation in most apoptosis cases [33]. As demonstrated by caspase activation assay and western blotting, caspases 9 and 3 activations in PA exposure were detected. The Bcl-2 family proteins play important roles in apoptosis [34]. Two main proteins of Bcl-2 family are the antiapoptotic Bax and the proapoptotic Bcl [35]. The unbalance of Bcl-2/Bax ratio contributes to the loss of MMP [36]. PA caused the unbalance of Bcl-2/Bax, collapse of MMP, and thus cascade of caspase pathway. MMP is an important converge point for many signaling intracellular apoptotic pathways [37]. Transcriptional factor NF-*κ*B plays an important role in the regulation of mitochondrial apoptotic pathway [38]. Many antitumor agents exert their ability through NF-*κ*B signaling pathway.

The antiproliferation effect of PA to A549 cell was weaker than SW480 cell but stronger than HCT116 cell* in vitro*. However, apparent antitumor effect of PA to A549 xenograft nude model was witnessed in our study* in vivo*. As previous report mentioned, PA induced two types of human colon cancer cell lines apoptosis via activating NF-*κ*B signaling pathway and downregulating c-myc [25]. Our result is associated with the published reports in mitochondrial apoptotic pathway. Furthermore, we conducted the cell cycle arrest assay in PA-treated A549 cell. In published report, PA treatment in HCT116 and SW480 cells activated P21 expression and suppressed the expressions of Cyclin D1 and Cyclin-dependent kinase 4 (CDK4) in a dose-dependent manner [25]. Similar to previous result, PA induced accumulating G_1_/S cell cycle arrest through activation in P53 and P21 and suppression in CDK2 and Cyclin E.

**Figure 5:**
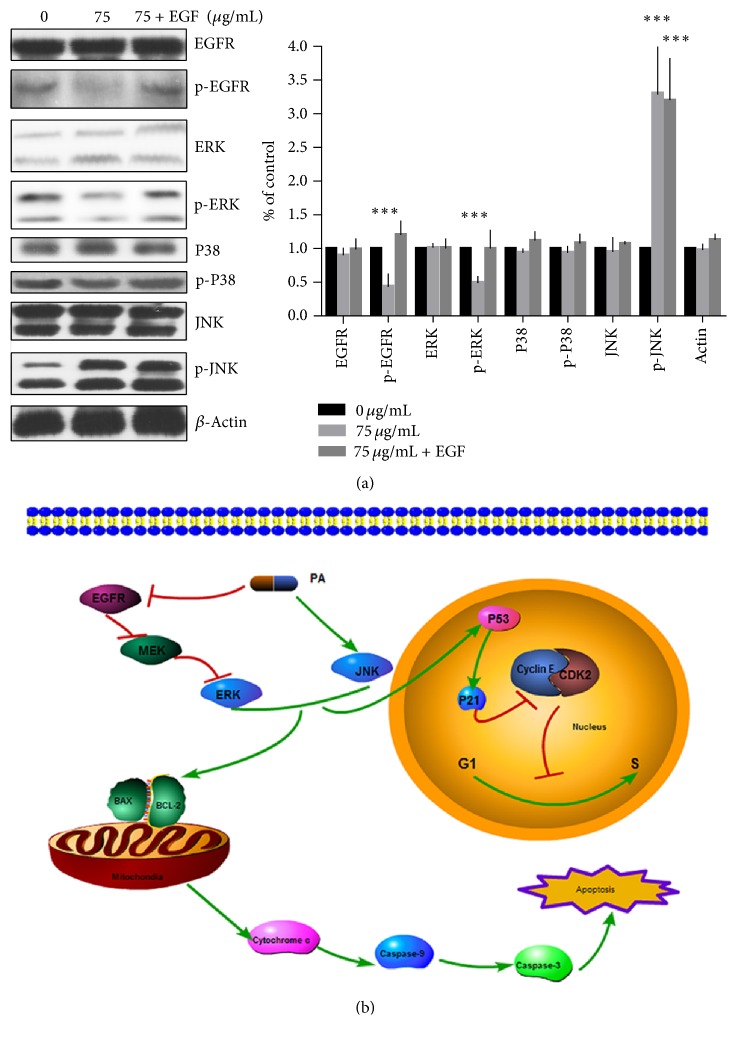
A549 cells were exposed for 48 h combined with EGF. (a) Mechanism certification of PA induced apoptosis and cell cycle arrest on A549 cells. (b) The graphical abstract of PA's action on A549 cells. Statistical differences were compared between PA treatment group and control group and considered significant at the levels of ^*∗∗∗*^ *P* < 0.001.

EGFR plays important roles in cell proliferation, differentiation, oncogenesis, and development [39]. The expression level of EGFR was commonly upregulated in many cancers, for instance, colon [40], ovarian [41], breast [42], and lung especially [43]. We conducted western blotting assay to certify apoptotic mechanism. We used the EGF (100 ng/mL) in activation of EGFR signal pathway [44]. The apoptosis in PA combined with EGF group was decreased compared with PA, while cell cycle distribution was altered, as shown in Figures 1(d) and 3(a). The EGFR phosphorylation levels were reupregulated in the combination group, as shown in Figure 5(a). Our results show that PA treatment significantly blocked the phosphorylation of EGFR and its downstream signaling: phosphorylation of MEK and ERK proteins. Importantly, exotic addition of EGF reactivated the downstream pathway of EGFR and reversed the apoptosis as well as cell cycle arrest induced by PA. This indicated that blocking the phosphorylation of EGFR is necessary for the cytotoxicity of PA.

In summary, we reported that PA induced apoptosis and cell cycle arrest in A549 cancer cells* in vitro* and* in vivo* through blocking phosphorylation of EGFR pathways and activating JNK pathways for the first time to our knowledge. PA also arrests G_1_/S cycle distribution through impact on CDK2/Cyclin E complex. The graphical mechanism of PA's action on A549 cells was demonstrated in Figure 5(b). Our findings suggested that PA may be a promising candidate for antitumor agent.

## Figures and Tables

**Table 1:** The inhibitory effect of PA on A549 implantation tumor growth in BALB/C-nu mice.

Groups (mg/kg)	Number of animals survived	Body weights (g)		Tumor weights (g)	Inhibition rate (%)
		Start	End		
Control	6	20.38 ± 0.66	24.78 ± 0.93	1.35 ± 0.18	
5	6	20.7 ± 0.44	24.48 ± 0.68	0.78 ± 0.11^*∗*^	42.35
10	6	20.67 ± 0.42	24.48 ± 1.43	0.51 ± 0.23^*∗∗∗*^	62.50
15	6	20.67 ± 0.41	23.38 ± 0.57	0.27 ± 0.19^*∗∗∗*^	79.70

^*∗*^ *P* < 0.05 versus the vehicle control.

^*∗∗∗*^ *P* < 0.001 versus the vehicle control.
